# Anti‐inflammatory properties of *ophioglonin* derived from the fern *Ophioglossum vulgatum* L. via inactivating NF‐κB and MAPK signaling pathways

**DOI:** 10.1002/2211-5463.13914

**Published:** 2024-10-25

**Authors:** Xiaoqing Zhu, Cheng Tian, Dan Yao, Siqi Li, Junjiang Lv, Yongwen Chen, Xiaoyong Huang

**Affiliations:** ^1^ Science and Technology Industry Development Center, Chongqing Medical and Pharmaceutical College Chongqing China; ^2^ Institute of Immunology, People's Liberation Army Third Military Medical University Chongqing China; ^3^ Chongqing Key Laboratory of High Active Traditional Chinese Drug Delivery System Chongqing Medical and Pharmaceutical College Chongqing China

**Keywords:** anti‐inflammation, antioxidant, macrophages, mechanism, Ophioglonin, *Ophioglossum vulgatum*

## Abstract

Medicinal plants contain bioactive compounds that have therapeutic effects on human health. *Ophioglossum vulgatum* L. is a representative species of the fern genus *Ophioglossum* that has anti‐inflammatory properties as recognized in folk medicine. Herein, we performed a nitric oxide (NO) assay‐guided screening in RAW264.7 cells to investigate the active components of the plant. We found that *ophioglonin* (OPN), a characteristic homoflavonoid of the genus *Ophioglossum*, is one of the bioactive components. Therefore, we performed a comparative analysis of the isolated compounds and found that OPN has effects similar to those of isolated dihydroquercetin and luteolin at the concentrations tested. The antioxidant and anti‐inflammatory activities of OPN were extensively validated using lipopolysaccharide ‐stimulated RAW264.7 cells, mouse bone marrow‐derived macrophages (BMDMs), and peritoneal exudate macrophages (PEMs). *In vivo* experiments with a carrageenan‐induced mouse paw edema model further confirmed the anti‐inflammatory effect of OPN. Additionally, we found that OPN and *Ophioglossum vulgatum* extracts inhibit the activation of signal transducers, NF‐ĸB p65, IĸBα, ERK, p38, and JNK, consistent with the findings of pathway enrichment analysis. This work reinforces the anti‐inflammatory properties of *Ophioglossum vulgatum* and indicates that OPN is a promising therapeutic agent for inflammation‐associated disorders. Further clinical evaluations, including clinical trials, would be beneficial to validate the anti‐inflammatory properties of OPN.

## Graphical abstract

Phytochemical analysis revealed that *ophioglonin* is a bioactive component involved in anti‐inflammatory effects of the fern *Ophioglossum vulgatum*. The anti‐inflammatory properties of *ophioglonin* were extensively validated in various models. *Ophioglonin* and *Ophioglossum vulgatum* extracts were found to inhibit NF‐ĸB and MAPK signaling pathways, consistent with the findings of enrichment analysis.

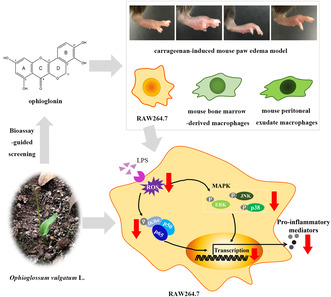

**Abbreviations**
3MQ: 3‐O‐methylquercetin
BMDMs: mouse bone marrow‐derived cells
BP: biological process
Ca: carrageenan
CC: cellular component
CCK8: cell counting kit‐8 assay
COX‐2: cyclooxygenase‐2
DCFH‐DA: 2,7‐dichlorodihydrofluorescein diacetate
DCM: dichloromethane
DHQ: dihydroquercetin
EA: ethyl acetate
ELISA: enzyme‐ linked Immunosorbent assay
ERK: extracellular signal‐regulated kinase
GO: gene ontology
HPLC: high‐performance liquid chromatography
HRP: streptavidin‐horseradish peroxidase
IL‐1β: interleukin‐1β
IL6: interleukin 6
Indo: indomethacin
*iNos*
: inducible nitric oxide synthase
JNK: Jun‐amino‐terminal kinase
KEGG: Kyoto encyclopedia of genes and genomes.
LPS: Lipopolysaccharides
MAPK: mitogen‐activated protein.
M‐CSF: macrophage colony‐stimulating factor
MF: molecular function
n‐BuOH: n‐butanol
NF‐ĸB: nuclear factor kappa B
NMR: nuclear magnetic resonance spectroscopy
NO: nitric oxide
*O. vulgatum*
: *Ophioglossum vulgatum* L.
OPN: ophioglonin
OVE: *Ophioglossum vulgatum* L. extract
PE: petroleum ether
PEMs: murine peritoneal exudate macrophages
qRT‐PCR: quantitative Real‐time PCR
ROS: reactive oxygen species
TLC: thin‐ layer chromatography
TNFα: tumor necrosis factor alpha

Inflammation is a biological response exhibited by the host against invading pathogens or endogenous stimuli [1, 2]. It is a protective mechanism for tissue repair or restoration from harmful signals. However, increasing evidence suggests that excessive and uncontrolled inflammation is implicated in various chronic diseases, including cancer, diabetes, rheumatic, cardiovascular, pulmonary, and inflammatory bowel diseases [3].

Previous studies have reported that macrophages are constantly activated as pro‐inflammatory M1 phenotype during chronic disease progression [4, 5, 6]. This leads to unchecked production of pro‐inflammatory mediators, including reactive oxygen species (ROS), cyclooxygenase‐2 (COX‐2) and inducible NO synthase (iNOS), nitric oxide (NO), interleukin 6 (IL6), IL‐1β and tumor necrosis factor alpha (TNFα) [7, 8, 9, 10], which results in an imbalance between the pro‐inflammatory and anti‐inflammatory signals, and uncontrolled inflammation and long‐lasting tissue damage [3, 4]. In response to inflammatory stimuli that activate macrophages, intracellular signaling pathways are activated to induce the production of pro‐inflammatory mediators. To date, the best‐studied canonical pathways are the nuclear factor kappa B (NF‐κB) signaling pathway and mitogen‐activated protein kinase (MAPK) pathways [11, 12, 13, 14]. Consequently, intervention approaches targeting these pathways may offer new therapeutic strategies against inflammation‐associated pathologies [15, 16, 17]. Previous studies reported that natural products and their isolated compounds, for example, *Boswellia serrata*, *Smilax glabra Roxb*., *Matricaria recutita* L., and *Persicaria chinensis* L., have anti‐inflammation properties by blocking NF‐κB and MAPK signaling pathways, enriching conventional therapies for treating diseases [18, 19, 20, 21, 22, 23].

*Ophioglossum vulgatum* L. (*O. vulgatum*) belongs to the ancient fern genus *Ophioglossum*, commonly known as adder's tongue because its fertile spike resembles a snake's tongue [24]. The genus is widespread in the temperate regions of the Northern Hemisphere, with different species reported in Europe and Asia [24, 25]. Historically, the genus *Ophioglossum* has been used in medicine, and records of its traditional applications have been discovered in several countries. In Italy, fresh fronds of *O. vulgatum* are used to make an olive oil ointment to treat skin wounds [26]. In England, it is known as the ‘green oil of charity’, and in China, the entire plant is known for its heat‐clearing and detoxifying effects [26]. Currently, pharmacological studies of the *Ophioglossum* species are rather scattered. A previous study of *O. vulgatum* reported that the galactoglycerolipids from the frond dichloromethane extract increased wound closure rates in HaCaT keratinocytes [26]. Another previous study on the species *O. thermale* Kom confirmed the antibacterial activity of two thermalic acids from the crude extracts [27]. Although a previous study reported that the ethyl acetate (EA) fraction of the species *O. thermale* exerted an anti‐inflammatory effect on carrageenan‐induced mouse hind paw edema [28], i the mechanism and components responsible for the anti‐inflammatory effect are unclear.

Consequently, this study aimed to investigate the active components and the mechanism of action of theanti‐inflammatory effects of *O. vulgatum* using an activity‐guided approach.

## Materials and methods

### Preparation of *O. vulgatum* extracts (OVE)

Fresh *O. vulgatum* L. were obtained from Guangan, Sichuan Province, China (106°42′ E, 30°67′ N) in November 2022. Permit was not required for raw material collection in this region. The materials were identified by Associate Professor Yu Dai from the department of Traditional Chinese Medicine, Chongqing Medical and Pharmaceutical College, China. A voucher specimen was stored in the laboratory under the code number YGZ1115OphV‐Scga. For OVE preparation, 12 kg of *O. vulgatum* were firstly kept in 95% ethanol for 3 weeks and then extracted for 2 h by reflux. The extracted solution was finally concentrated and dried under reduced pressure, yielding 160 g dry solid extracts.

### RAW264.7 cell culture

Murine macrophage cells RAW264.7 were kindly provided by Professor Yongwen Chen from Third Military Medical University. Cells were cultured in Dulbecco's modified Eagle's medium (DMEM) medium (Gibco™, Thermo Fisher Scientific, Waltham, MA, USA) supplemented with 10% fetal bovine serum (FBS; Gibco™, Thermo Fisher Scientific) and 1% penicillin–streptomycin (Gibco™, Thermo Fisher Scientific). Cells were incubated in a sterile incubator supplied with 5% CO_2_ and kept at 37 °C. Cells were subcultured every 2 days.

### Bioassay‐guided screening of highly active constituents from OVE

**Fig. 1:**
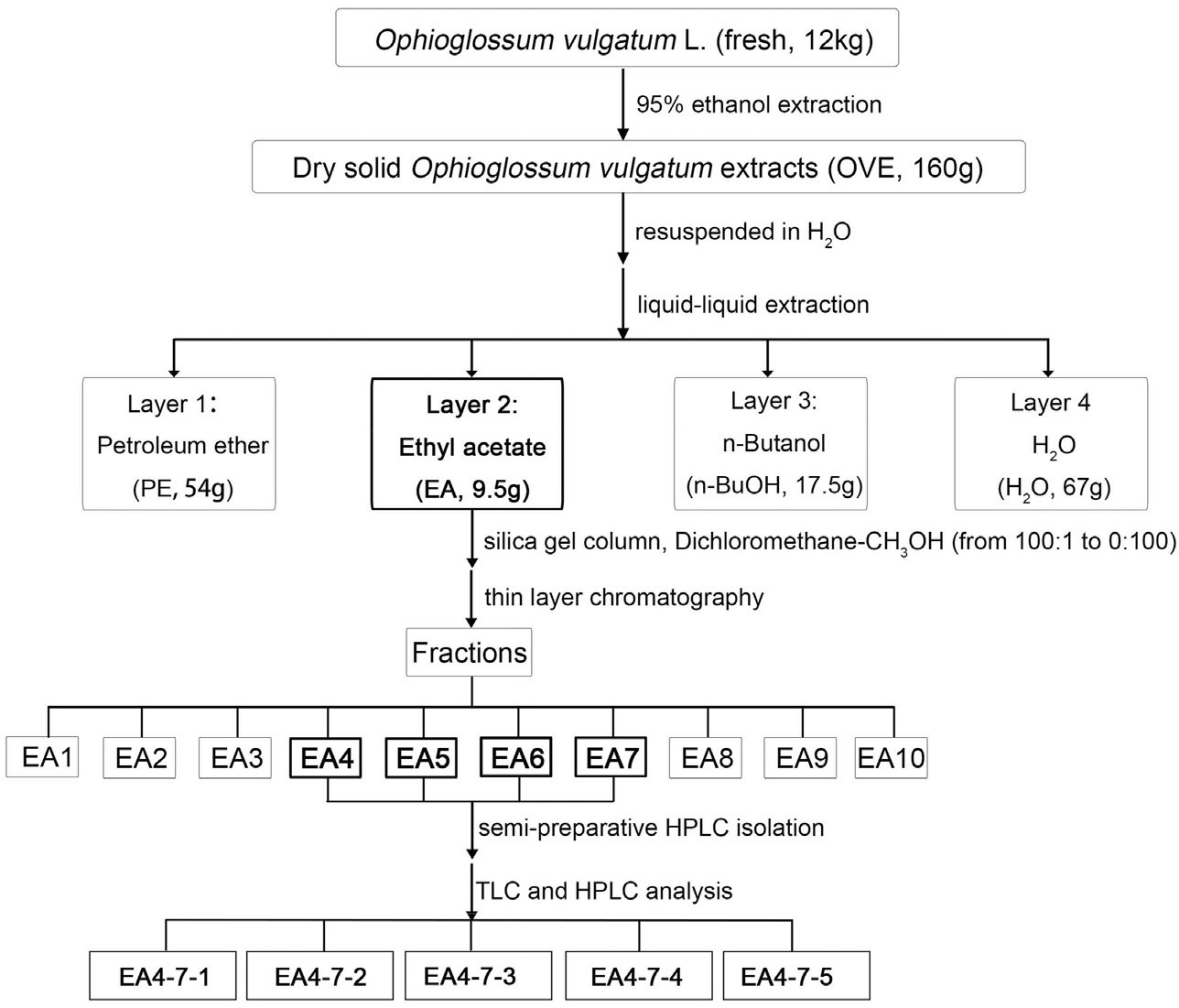
Bioassay‐guided screening of highly active constituents from OVE.

The workflow of the screening process was presented in Fig. 1. For initial partition, the dry OVE were firstly resuspended in H_2_O and then extracted by different polar solvents, petroleum ether (PE), EA, and n‐butanol (n‐BuOH). For cell treatment, extracts from each fraction were dissolved in DMSO and cell culture medium and then diluted to the indicated concentrations for NO assay detection in RAW264.7 cells. In alignment with the previous reports, EA layer was identified as the highly active part to inhibit LPS‐induced NO production.

Next, bioactive EA layer was subjected to open silica gel column chromatography and gradient mixtures of dichloromethane (DCM)‐CH_3_OH (from 100:1 to 0:100) were used as eluents. Fractions were combined according to similar thin‐layer chromatography (TLC) results, and 10 final fractions were obtained for bioactivity screening. Subsequently, fractionsEA4‐7 were recognized with high bioactivity and were further separated by semi‐preparative high‐performance liquid chromatography (semi‐preparative HPLC; LC3050N Type; Beijing Chuangxintongheng Science and Technology Co., Ltd, Beijing, China). C18 column (30 × 150 mm, with 5 mm particle size; Agela Technologies, Tianjin, China) was used for separations with mobile phase consisting of 40% H_2_O and 60% CH_3_OH. The flow rate was 15 mL·min^−1^. As a result, five compounds were isolated based on TLC and HPLC analysis.

Finally, all the five isolated compounds were crystallized and were then sent for identification by nuclear magnetic resonance (NMR) spectrometry analysis (600 MHz, Agilent Technologies, Santa Clara, CA, USA) in Innovative Drug Research Center of Chongqing University.

### Viability assay

RAW264.7 cells were counted and seeded in 96‐well plate (30 000 cells·well^−1^) 4 h before the treatment. Cells were treated with OVE or compounds dissolved at different concentrations with or without LPS (100 ng·mL^−1^; Sigma‐Aldrich, Saint Louis, MO, USA). DMSO was used for the untreated control groups. After 24 h incubation, Cell Counting Kit‐8 assay (CCK8; Dongren Chemical Technology, Shanghai, China) were performed to detect cell proliferation. The 450 nm optical density was measured, and cell viability in percentage was calculated for each condition. The experiments were performed in triplicates.

### ROS level detection

RAW264.7 cells were seeded in 12‐well with a density of 500 000 cells·well^−1^. After 4 h incubation, cells were pretreated with OVE or OPN at indicated concentrations and DMSO was used in the control groups. In the following, LPS (100 ng·mL^−1^) was added to the medium in selected wells for stimulation. After 24 h incubation, ROS levels were detected by following the instruction manual of the ROS Assay Kit (Beyotime, Beijing, China). In brief, DCFH‐DA probe diluted in prewarmed DMEM medium (1:1000) was added to selected wells and cells were further incubated in the incubator for 25 min. Cells were then washed twice with prewarmed DMEM medium and collected in PBS for fluorescence detection by Flow cytometer BD FACSCanto II (BD, Franklin Lakes, NJ, USA). The experiments were performed in triplicates.

### Nitric oxide (NO) assay

RAW264.7 cells were seeded in 24‐well with a density of 250 000 cells·well^−1^. After 4 h incubation, cells were pretreated with OVE, fractions, or components dissolved at different concentrations for 1 h and DMSO was used for control groups. In the following, LPS (100 ng·mL^−1^) was added to the medium in selected wells for stimulation. After 24 h incubation, the medium of each well was collected and quantification of NO was performed by following the instruction manual of the manufacturer (Beyotime). Briefly, Griess Reagents I and II were firstly recovered to room temperature for 30 min. For the measurement, 50 μL of the medium was added to a 96‐well plate and 50 μL of Griess Reagents I and II was added successively. Finally, the 540 nm optical density was detected and NO production was calculated for each condition. The experiments were performed in triplicates.

### Enzyme‐linked immunosorbent assay (ELISA)

Cells were treated as described in subsection 2.5. The cell culture supernatant of each well was collected and measurement of TNFα, IL‐1β, and IL‐6 secretion was performed by following the instructions of enzyme‐linked Immunosorbent assay (ELISA) kits (Boster, Wuhan, China). Briefly, a 96‐well plated precoated with capture antibody was used for detection. Firstly, 100 μL of the cell culture supernatant or standard solution was added into the precoated wells and incubated at 37 °C for 1.5 h. After rinsing the wells, 100 μL of working solution containing biotinylated antibody was added to each well and again incubated at 37 °C for 1 h. After that, the wells were washed for 4 times to remove the excess detection antibody. Subsequently, streptavidin–horseradish peroxidase (HRP) working solution was added and the plate was incubated at 37 °C for 30 min. After rinsing the wells again, 100 μL of tetramethylbenzidine reagent solution was added into each well and incubated at 37 °C in the dark for 10–30 min. Subsequently, 100 μL of stop solution was added and the absorbance at 450 nm was measured. Cytokine production was determined by using the standard curves of the respective standard reagents in each ELISA kit. The experiments were performed in triplicates.

### Quantitative real‐time PCR (qRT‐PCR)

Cells were treated as described in subsection 2.5. Total RNA for each condition was isolated according to the manufacturer's instructions of TRIzol reagent (Takara, Dalian, China). For qRT‐PCR analysis, RNA was firstly reversely transcribed into complementary DNA with a reverse transcription kit (Takara). For quantification of gene expression, comparative CT method (ΔΔCT) was adopted by using gene‐specific primers for amplification. The primers were as listed in Table 1.

**Table 1:** Primer list used in this study.

Gene name	Primer sequence (5′‐3′)
*iNos*	5′‐GAGACAGGGAAGTCTGAAGCAC‐3′
	5′‐CCAGCAGTAGTTGCTCCTCTTC‐3′
*Il6*	5′‐TACCACTTCACAAGTCGGAGGC‐3′
	5′‐CTGCAAGTGCATCATCGTTGTTC‐3′
*Il‐1b*	5′‐TGGACCTTCCAGGATGAGGACA‐3′
	5′‐GTTCATCTCGGAGCCTGTAGTG‐3′
*Tnfα*	5′‐GGTGCCTATGTCTCAGCCTCTT‐3′
	5′‐GCCATAGAACTGATGAGAGGGAG‐3′
*Cox‐2*	5′‐GCGACATACTCAAGCAGGAGCA‐3′
	5′‐AGTGGTAACCGCTCAGGTGTTG‐3′
*Gapdh*	5′‐CATCACTGCCACCCAGAAGACTG‐3′
	5′‐ATGCCAGTGAGCTTCCCGTTCAG‐3′

### Mice

Male C57BL/6 mice, approximately 6 weeks old, were purchased from the Jackson laboratory (Bar Harbor, ME, USA). All mice were housed in the animal center of Third Military Medical University (TMMU) and received humane care according to the Guide for the Care and Use of Laboratory Animals. In detail, mice were kept in microisolator cages at room temperature (22–25 °C) with humidity of 50–60% and provided with unlimited access to standard diet and water on a 12 h light/12 h dark cycle. The physical condition of the experimental mice was observed every day, including water and food intake and the mental state. None of the experimental mice became severely ill or died prior to the experimental endpoint. After 1‐week acclimation, mice were proceeded for experiments and all protocols were approved by the Laboratory Animal Welfare and Ethics Committee of TMMU (no. SYXK‐PLA‐20220018).

### Primary murine macrophages isolation, culture, and treatment

#### Peritoneal exudate macrophages (PEMs)

To obtain elicited peritoneal macrophages, 1 mL 3% Thioglycollate broth (Becton, Dickinson and Company, Sparks, MD, USA) was injected into the peritoneal cavity of each mouse for 3 days. Mice were euthanized by rapid cervical dislocation on day 4. After soaking each mouse within 70% ethanol for 1–2 min, the peritoneal exudate cells from 3 mice were collected with DMEM medium (Gibco™, Thermo Fisher Scientific) lavage and kept on ice. Cells were centrifuged at 800 rpm for 5 min, and the supernatant was discarded. Red blood cells lysis buffer (Beyotime) was then utilized for purification, and cells were centrifuged again for collection. Finally, cells were resuspended in DMEM culture medium supplemented with 10% FBS and seeded in a 12‐well plate. After 4 h incubation at 37 °C, supernatant in each well was removed and cells attached to the bottom were recognized as PEMs. In the following, PEMs were pretreated with OVE or compounds at indicated concentrations for 1 h and LPS (100 ng·mL^−1^) was added to the medium in selected wells for stimulation. Cell culture supernatant, total RNA, and protein of each well were collected at different time points for various assays. All the experiments were performed in triplicates.

#### Bone marrow‐derived macrophages (BMDMs)

Mice were euthanized by rapid cervical dislocation. After the skin being peeled from the top of each hind leg, both hind legs of each mouse were cut off at the hip joint in sterile condition. Muscles on the legs were removed, and intact femurs were placed in plastic dish with sterile DMEM medium. By inserting the needle into the bone marrow cavity of each femur, bone cavity was flushed several times with medium until it turned to white. Finally, all the wash media were collected in a sterile tube and centrifuged at 800 rpm for 5 min. Supernatant was then discarded and red blood cell lysis buffer was added for purification. Cells were then centrifuged again for collection, and the pellet was resuspended in complete culture medium consisting of DMEM medium, 10% FBS, and 50 ng·mL^−1^ macrophage colony‐stimulating factor (M‐CSF; MCE, Monmouth Junction, NJ, USA) for macrophage induction. Cells were seeded in 6‐well plate and incubated at 37°C for 6 days. Medium of each well was changed on day 3 with complete culture medium and on day 5 with reduced M‐CSF (10 ng·mL^−1^). Finally, BMDMs were treated on day 6 as described in Section 2.10.1. Cell culture supernatant, total RNA, and protein of each well were collected at different time points for various assays. All the experiments were performed in triplicates.

### Carrageenan‐induced mouse paw edema model

Mice were randomly divided into four groups as Control, 1% Carrageenan (1% Ca), 1% Carrageenan + Indomethacin (1% Ca + Indo), and 1% Carrageenan + Ophioglonin (1% Ca + OPN), with 5 mice in each group. The positive group (1% Ca + Indo) was pretreated with 10 μg·g^−1^ indomethacin (MCE) by intraperitoneal (i.p.) injection for 45 min; the test group (1% Ca + OPN) was pretreated with 40 μg·g^−1^ ophioglonin by i.p. injection for 45 min; the control group and 1% Ca group were given PBS (Gibco™, Thermo Fisher Scientific). To induce paw edema, 50 μL of 1% Carrageenan (Solarbio, Beijing, China) was injected into the left hind paw (i.h.) of each mouse in the three groups of 1% Ca, 1% Ca + Indo, and 1% Ca + OPN; the control group was given 50 μL PBS as control. The measurements were performed at 0, 2, 4, 6, 10, and 24 h after carrageenan injection using vernier caliper. The inhibitory effect was determined by the thickness of paw edema. No animals died during the experiments. At the end of the experiment, mice were expertly euthanized by cervical dislocation method according to the approved protocols.

### Gene ontology (GO) and kyoto encyclopedia of genes and genomes (KEGG) analysis for common genes between OPN and inflammation

The PharmMapper database (https://lilab‐ecust.cn/pharmmapper/index.html) and SwissTargetPrediction webtool (http://swisstargetprediction.ch/) were employed to predict target genes for OPN. Genes with Norm Fit >0.5 were screened from PharmMapper. A total of 441 genes were finally collected as potential OPN targets by eliminating replicates.

Inflammation‐associated genes were acquired from databases: GeneCards (https://www.genecards.org/) and OMIM (https://www.omim.org/) by searching for the key word ‘inflammation’. Genes with relevance ≥10 were screened from GeneCards. Identical genes were removed, and 1023 genes were finally collected as inflammation‐associated targets.

The Venny 2.1.0 (https://bioinfogp.cnb.csic.es/tools/venny/) was used to obtain the common genes between OPN and inflammation. The list of OPN–inflammation correlated genes was uploaded to the DAVID database (https://david.ncifcrf.gov/summary.jsp) for GO and KEGG pathway enrichment analysis. Biological process (BP), cellular component (CC), and molecular function (MF) of GO analysis were illustrated. Results of inflammation‐related KEGG pathways were presented.

### Western blotting

Cells treated with OVE or OPN were washed with cold PBS buffer and lysed in RIPA buffer (Beyotime). Protein concentration was detected by BCA assay (Thermo Fisher Scientific). Denatured at 95 °C for 5 min, samples were loaded into SDS/PAGE gels and transferred to 0.45 μm PVDF membranes (Millipore, Sigma‐Aldrich, Saint Louis, MO, USA). After blocked in 5% nonfat dry milk at room temperature for 1 h, membranes were incubated with primary antibodies at 4 °C on a shaker overnight and antibodies were listed in the following: anti‐COX‐2 (A00084‐2; Boster, Wuhan, China; 1:1000), anti‐IL6 (ab259341; Abcam, Cambridge, UK; 1:1000), anti‐IL‐1β (A00101‐4; Boster), anti‐TNFα (sc‐52 746; Santa cruz, Texas, USA; 1:200), anti‐phospho‐NF‐ĸB p65 (82335‐1‐RR; Proteintech, Wuhan, China; 1:500), anti‐NF‐ĸB p65 (A00284‐1; Boster), anti‐phospho‐IĸBα (MA01139; Boster), anti‐IĸBα (BM3932; Boster), anti‐β‐Actin (81115‐1‐RR; Proteintech, Wuhan, China; 1:1000), anti‐ERK (BM4326; Boster), anti‐phospho‐ERK (BM4156; Boster), anti‐phospho‐p38 (4511S; Cell Signaling Technology, Beverly, MA, USA; 1:1000), anti‐p38 (8690S; Cell Signaling Technology; 1:1000), anti‐JNK(BM4329; Boster; 1:500), and anti‐phospho‐JNK (BM4380; Boster). The blots were then incubated with corresponding secondary antibodies (Beyotime) for 1 h at room temperature and washed in tris buffered saline with 1‰ Tween‐20 (TBST) for three times. The results were finally detected by using ChemiDoc imaging system (Bio‐Rad, Hercules, CA, USA) or FUSION Solo S system (Vilber Bio Imaging, Beijing, China). The experiments were performed in triplicates.

### Statistical analysis

GraphPad Prism software (version 7.0; La Jolla, CA, USA) was used for statistical analysis. All data in this study were performed in triplicates and presented as the mean ± standard deviation (SD). Data were analyzed by using one‐way ANOVA analysis and Dunnett's *post hoc* test was adopted for multiple comparisons. Changes with *P*‐value <0.05 were identified as statistical significance.

## Results

### Antioxidant and anti‐inflammatory activities of *O. vulgatum* extracts (OVE) in lipopolysaccharide (LPS)‐stimulated RAW264.7 cells

**Fig. 2:**
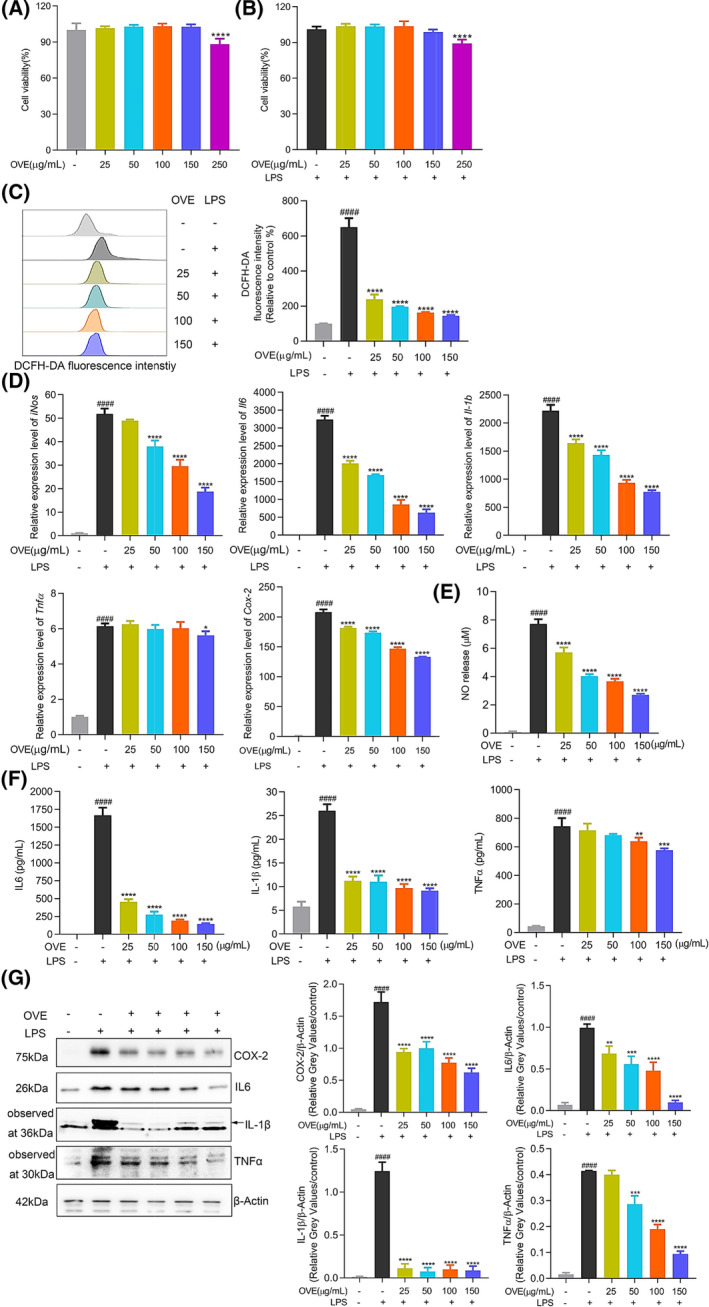
Antioxidant and anti‐inflammatory effects of OVE in LPS‐stimulated RAW264.7 cells. (A, B) Cell viability measured by CCK8 assay. (C) Analysis of ROS levels detected by DCFH‐DA probe. (D) Quantitative analysis of gene expression levels of *iNos*, *Il6*, *Il‐1b*, *Tnfα*, and *Cox‐2* by qRT‐PCR. (E) NO production analysis by NO assay. (F) ELISA results of IL6, IL‐1β, and TNFα. (G) Protein expression levels of COX‐2, IL6, IL‐1β, and TNFα. All experiments were carried out in triplicates and data are presented as means ± SDs; one‐way ANOVA analysis was adopted for multiple comparisons; ^####^ *P* < 0.0001, compared to the untreated control group; *P < 0.05, **P < 0.01, ***P < 0.001, and ****P < 0.0001, compared to the LPS control group.

Initially, we assessed the cytotoxicity of OVE in RAW 264.7 cells to determine the concentrations of OVE used in the anti‐inflammation analysis. Cell viability analysis (Fig. 2A,B) revealed no toxicity at 25, 50, 100, and 150 μg·mL^−1^ concentrations used in the subsequent experiments. After 1 h pretreatment with OVE, production levels of multiple pro‐inflammatory mediators were analyzed following 24 h of LPS stimulation. As shown in Fig. 2C, OVE treatment dramatically decreased the intracellular ROS levels induced by LPS. Furthermore, qRT‐PCR results in Fig. 2D demonstrated that OVE inhibited *iNos*, *Il6*, *Il‐1b*, *Tnfα*, and *Cox‐2* gene expression in a dose‐dependent manner. The following assays demonstrated that OVE treatment significantly suppressed the production levels of NO, IL6, IL‐1β, TNFα, and COX‐2 proteins (Fig. 2E–G), further confirming the anti‐inflammatory effect of OVE.

### Identification and analysis of ophioglonin (OPN) as one of the highly active components from *O. vulgatum* extracts

#### Identification of OPN

**Fig. 3:**
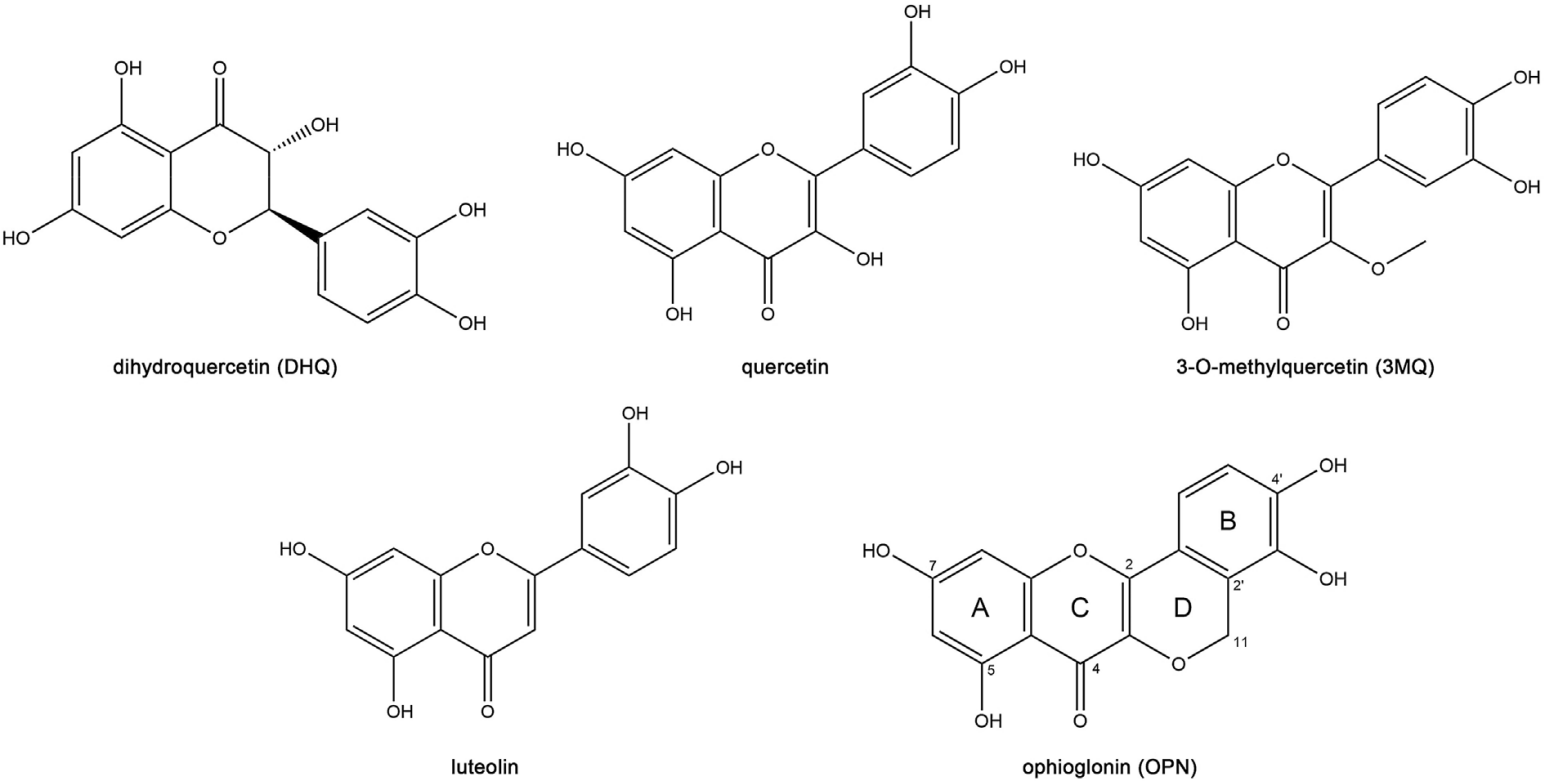
Chemical structures of all the five isolated compounds. Dihydroquercetin (DHQ), molecular formula: C_15_H_12_O_7_, purity 99%; quercetin, molecular formula: C_15_H_10_O_7_, purity 98%; 3‐*O*‐methylquercetin (3MQ), molecular formula: C_16_H_12_O_7_, purity 99%; luteolin, molecular formula: C_15_H_10_O_6_, purity 98%; ophioglonin (OPN), molecular formula: C_16_H_10_O_7_, purity 99%.

Following the NO assay‐guided screening (Fig. 1), we isolated five highly active compounds with ≥98% purity and used NMR analysis for compound identification. The ^13^C‐NMR and ^1^H‐NMR spectra data were obtained and compared with published data for characterization. The five isolated components were annotated as dihydroquercetin (DHQ, EA4‐7‐1) [29], quercetin (EA4‐7‐2) [30], 3‐*O*‐methylquercetin (3MQ, EA4‐7‐3) [31], luteolin (EA4‐7‐4) [32], and ophioglonin (OPN, EA4‐7‐5) [33]. The chemical structures of all the five compounds were illustrated in Fig. 3.

#### Comparative analysis of the effects of OPN on NO production and cell viability with the other isolated compounds

OPN is characterized as a typical member of the type II homoflavonoids, which is abundant in the genus *Ophioglossum*. The anti‐inflammatory effects of OPN on inflammation remain largely unknown compared to the isolated common flavonoids (DHQ, quercetin, 3MQ, and luteolin).

**Fig. 4:**
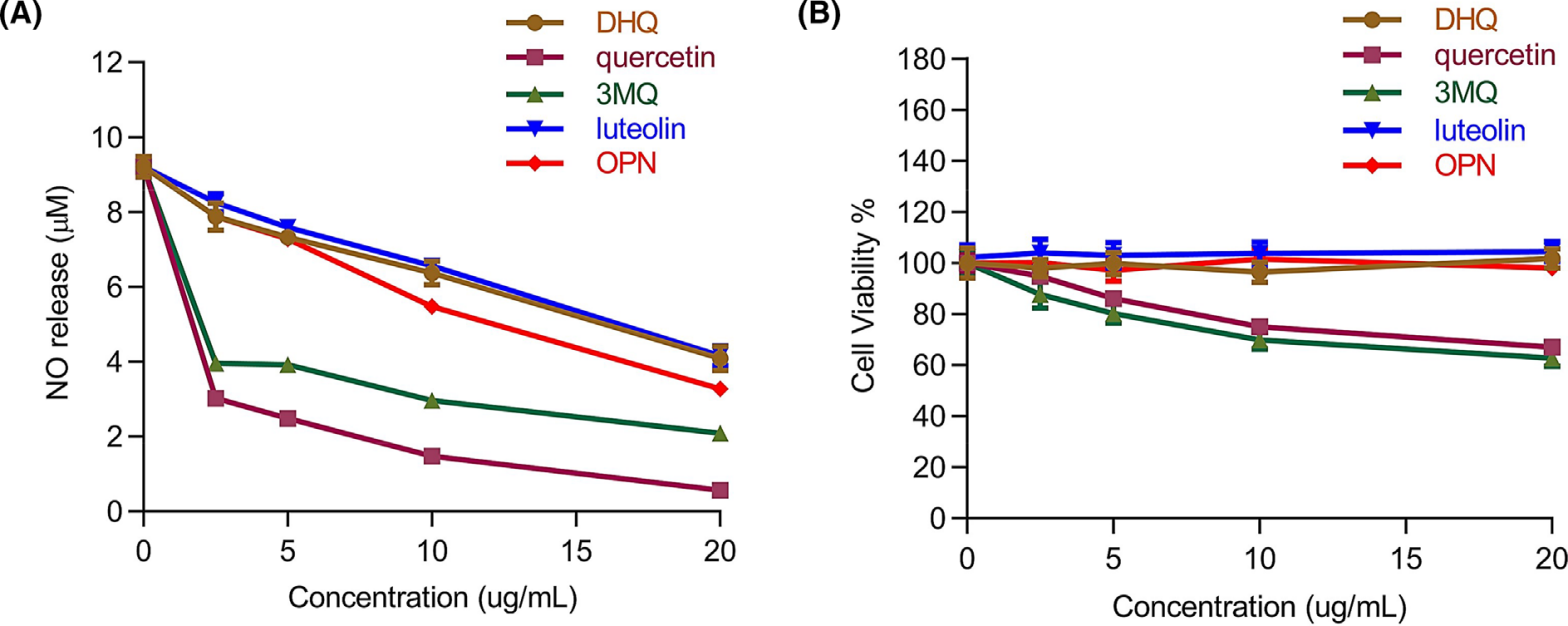
Comparative analysis of effects of isolated compounds on NO production and cell cytotoxicity. Cells were pretreated with isolated compounds at different concentrations for 1 h, and LPS (100 ng·mL^−1^) was added for 24 h stimulation. (A) NO production analysis by NO assay. (B) Cell viability measured by CCK8 assay. All experiments were carried out in triplicates, and data are presented as means ± SDs.

As a first insight into the anti‐inflammatory activity of OPN, we initially compared the effect of OPN on LPS‐induced NO production with the known isolated compounds. As shown in Fig. 4A, OPN exhibited a similar inhibitory effect with DHQ and luteolin on NO release. Although quercetin and 3MQ exhibited higher activities, cytotoxicity of these two compounds was detected at the concentrations tested in Fig. 4B.

### Antioxidant and anti‐inflammatory effects of OPN in LPS‐stimulated RAW264.7 cells

**Fig. 5:**
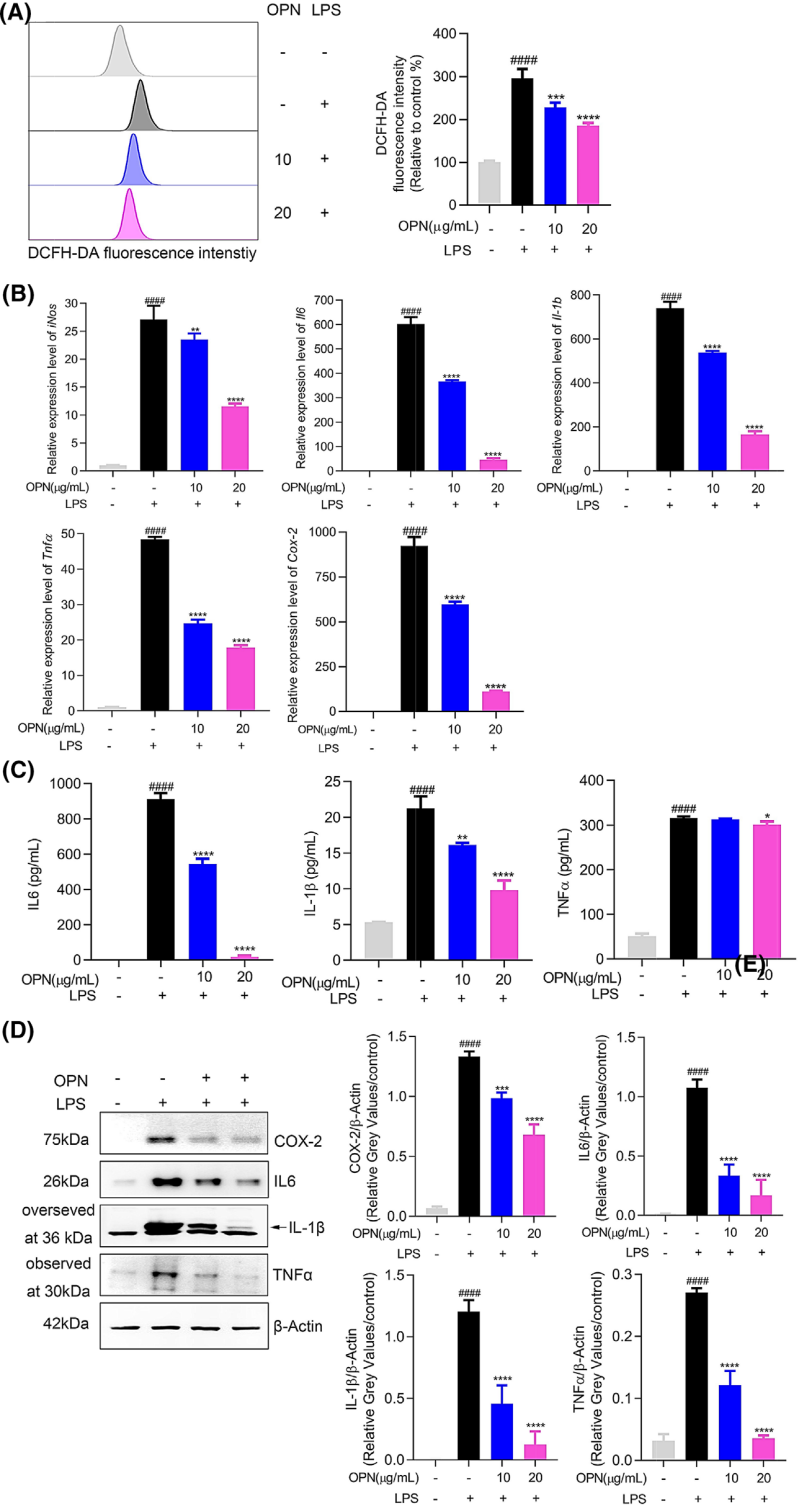
Antioxidant and anti‐inflammatory effects of OPN in LPS‐stimulated RAW264.7 cells. (A) Analysis of ROS levels detected by DCFH‐DA probe. (B) Quantitative analysis of gene expression levels of *iNos*, *Il6*, *Il‐1b*, *Tnfα*, and *Cox‐2* by qRT‐PCR. (C) ELISA results of IL6, IL‐1β, and TNFα. (D) Protein expression levels of COX‐2, IL6, IL‐1β, and TNFα. All experiments were carried out in triplicates and data are presented as means ± SDs; one‐way ANOVA analysis was adopted for multiple comparisons; ^####^ *P* < 0.0001, compared to the untreated control group; *P < 0.05, **P < 0.01, ***P < 0.001, and ****P < 0.0001, compared to the LPS control group.

Next, we comprehensively investigated the antioxidant and anti‐inflammatory activities of OPN. Production levels of multiple pro‐inflammatory mediators in RAW264.7 cells were analyzed after 24 h of LPS stimulation. As shown in Fig. 5A, OPN can reduce intracellular ROS levels. As demonstrated in Fig. 5B, OPN exerted an inhibitory effect on *iNos*, *Il6*, *Il‐1b*, *Tnfα*, and *Cox‐2* gene expression levels. Finally, results in Fig. 5C,D demonstrate the suppressive function of OPN on the production levels of IL6, IL‐1β, TNFα, and COX‐2 proteins.

### Effects of OPN on the production of pro‐inflammatory mediators in LPS‐stimulated primary murine macrophages

**Fig. 6:**
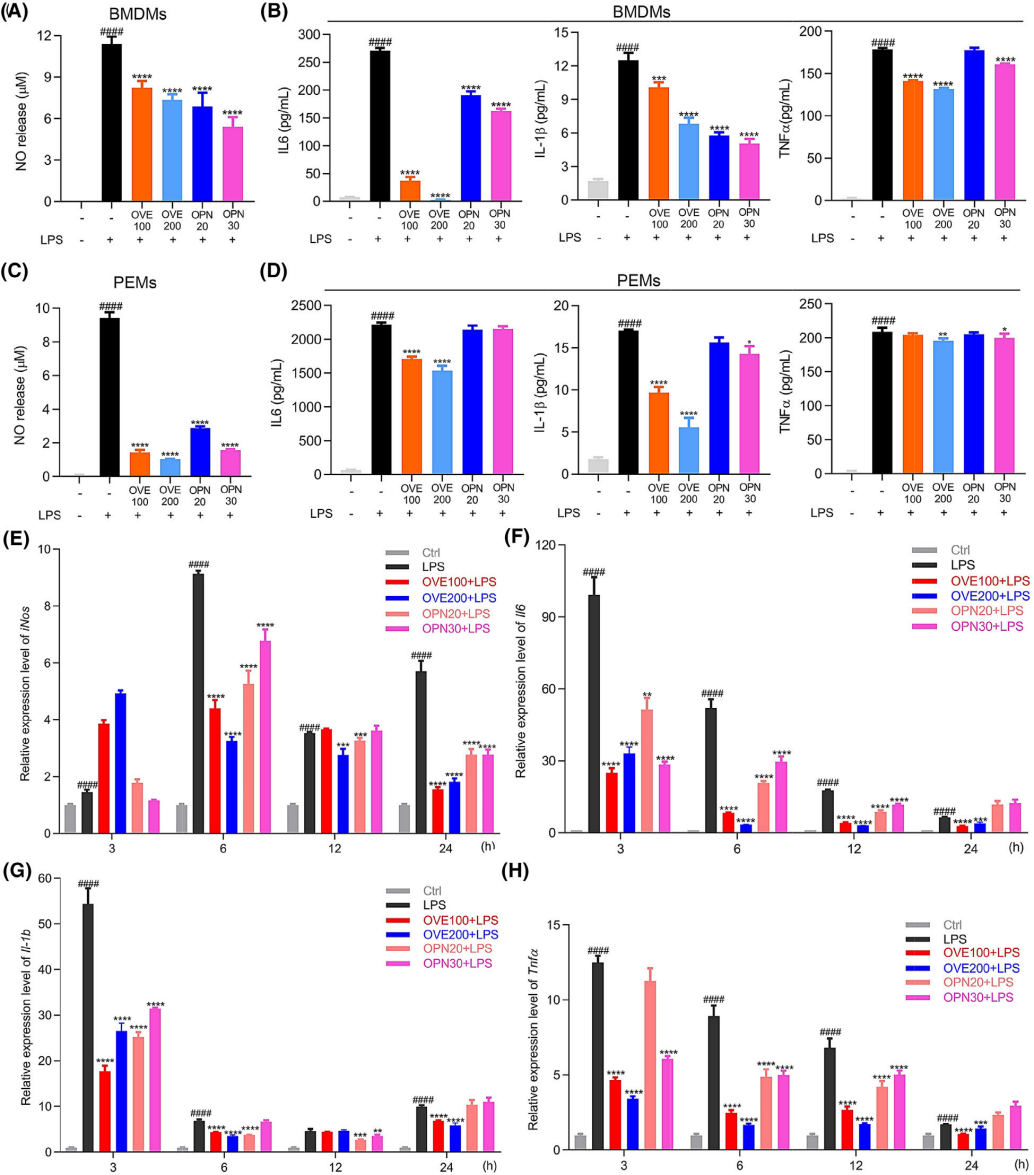
Effects of OVE and OPN on production of pro‐inflammatory mediators in primary mouse macrophages. After 1 h pretreatment of OPN (20 μg·mL^−1^, OPN20 and 30 μg·mL^−1^, OPN30) or OVE (100 μg·mL^−1^, OVE100 and 200 μg·mL^−1^, OVE200), primary mouse macrophages were treated with LPS (100 ng·mL^−1^) for 24 h stimulation in selected wells. (A, C) NO production in the cell culture supernatant detected by NO assay in BMDMs (A) and PEMs (C). (B, D) ELISA results of IL6, IL‐1β, and TNFα in BMDMs (B) and PEMs (D). (E–H) Time course of OVE and OPN effects on gene expression of pro‐inflammatory mediators in PEMs. After LPS (100 ng·mL^−1^) added for inflammation stimulation, total RNA of each condition was collected at different time points (3, 6, 12, and 24 h) and quantification of *iNos* (E), *Il6* (F), *Il‐1b* (G), and *Tnfα* (H) gene expression levels were measured. All experiments were carried out in triplicates and data are presented as means ± SDs; one‐way ANOVA analysis was adopted for multiple comparisons; ####*P* < 0.0001, compared to the untreated control group. Decreased expression levels with OPN and OVE treatment were analyzed for significance evaluation; *P < 0.05, **P < 0.01, ***P < 0.001 and ****P < 0.0001, compared to the LPS control group.

To further assess the anti‐inflammatory effects of OPN in primary mouse cells, we collected macrophages differentiated from mouse bone marrow‐derived cells (BMDMs) and murine macrophages from the thioglycollate‐stimulated peritoneal cavity (peritoneal exudate macrophages [PEMs]). Because of the heterogeneity of primary cells, pretreatment of OPN (20 and 30 μg·mL^−1^) and OVE (100 and 200 μg·mL^−1^) were used, and no significant cytotoxicity was observed in these cells. Production of NO and pro‐inflammatory cytokines IL6, IL‐1β, and TNFα were consequently examined after 24 h of LPS stimulation. The results of NO and ELISA assays confirmed the anti‐inflammation activities of OVE and OPN in primary murine macrophages (Fig. 6A–D).

As observed above, the inhibitory effect of OPN on pro‐inflammatory cytokines was remarkably mild in PEMs 24 h after LPS treatment. To further ascertain the inhibitory effect of OPN, we performed a time course of treatment and examined different gene expression levels. It was observed that the inhibitory effect of OPN was maintained at all the time points (3, 6, 12, and 24 h) in BMDMs (data not shown). However, in PEMs, the suppressive effects of OPN on *Il6*, *Il‐1b*, and *Tnfα* gene expression levels were observed to be restored after 24 h as shown in Fig. 6F–H, which may indicate a shorter elimination half‐life of OPN in this model.

### Effect of OPN on carrageenan‐induced mouse paw edema

**Fig. 7:**
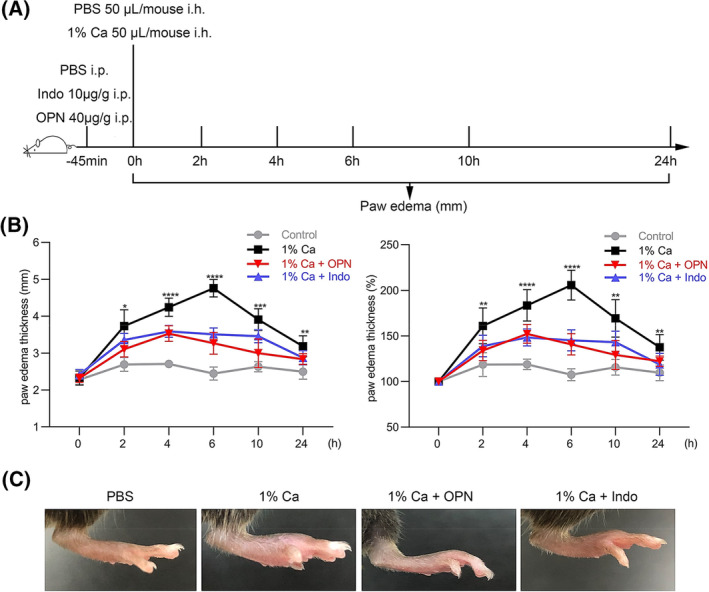
Effect of OPN on carrageenan‐induced paw edema. (A) Graphic experiment schedule. OPN (40 μg·g^−1^) was intraperitoneally (i.p.) injected in mice for 45 min, and 1% carrageenan (1% Ca) was then injected into the left hind paw (i.h.). Paw edema was measured at indicated time points. Indomethacin (Indo, 10 μg·g^−1^) was adopted as positive control. PBS was used in the untreated control groups. (B) Paw edema measurement. Data are presented as means ± SDs, *n* = 5; one‐way ANOVA analysis was adopted for multiple comparisons; *P < 0.05, **P < 0.01, ***P < 0.001 and ****P < 0.0001, for 1% Ca + OPN group compared to 1% Ca group. (C) Representative images of the left hind paw of mouses in four groups. Images were obtained at 6 h after carrageenan injection.

To further explore the anti‐inflammation property of OPN *in vivo*, we used the carrageenan‐induced paw edema model, and the experiment schedule was described in Fig. 7A. Injection of carrageenan (Ca) in the mouse paw caused an increase in the paw thickness (Fig. 7B), which peaked at 6 h and reduced after that. Indomethacin (Indo) at the concentration of 10 μg·g^−1^ of body weight was used as a positive control, demonstrating an inhibitory effect on the paw edema as reported. Based on preliminary observation in PEMs, OPN pretreatment was used at the concentration of 40 μg·g^−1^ of body weight for 45 min. The results demonstrated that OPN treatment led to a thinner paw with less swelling at 6 h (Fig. 7C), and substantially, the paw thickness was reduced at all time points (2, 4, 6, 10, and 24 h).

### Mechanisms of action underlying the anti‐inflammatory effects of OPN

#### GO and KEGG enrichment analysis of common targets between OPN and inflammation

**Fig. 8:**
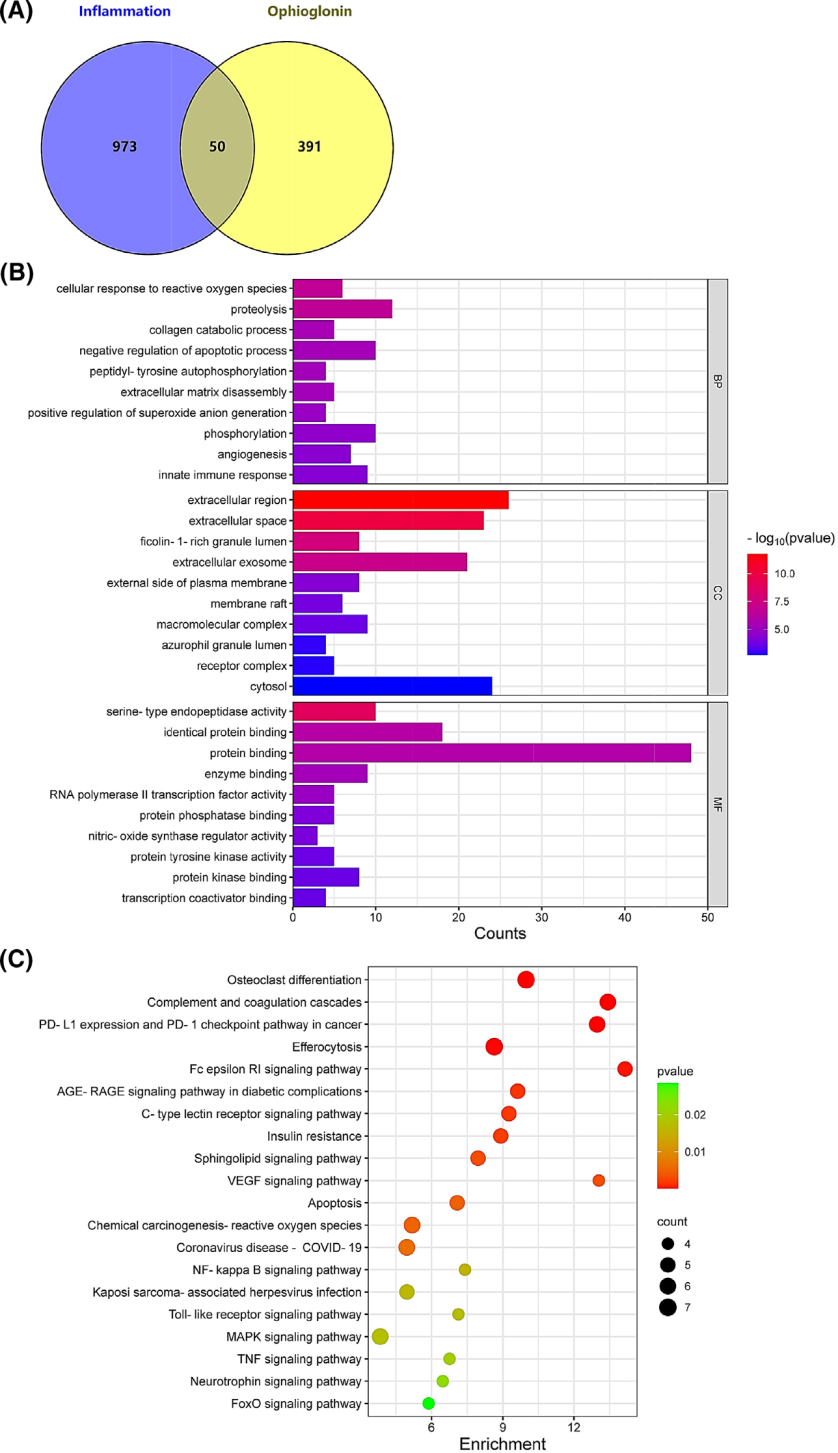
GO and KEGG enrichment analysis of common targets between OPN and inflammation. (A) Venn diagram of OPN targets (yellow circle) and inflammation‐associated genes (purple circle). (B) GO enrichment analysis. BP, biological processes; CC, cellular components; MF, molecular functions. (C) KEGG enrichment analysis. Top 20 inflammation‐associated pathways with lower *P*‐values were presented.

To explore the potential regulatory mechanisms for the anti‐inflammatory properties of OPN, the 441 OPN targets were clustered with 1023 inflammation‐associated genes and 50 common targets were obtained as shown in Fig. 8A. We uploaded the gene list to the DAVID database to perform GO and KEGG enrichment analyses which revealed that the cellular response to ROS, proteolysis, extracellular region, extracellular space, serine‐type endopeptidase activity, and protein binding were essential (Fig. 8B). Meanwhile, the top 20 inflammation‐related KEGG pathways annotation in Fig. 8C indicated that NF‐ĸB, Toll‐like receptor and MAPK signaling pathway may play vital roles in the inhibitory effect of OPN on inflammation.

#### Effects of OPN on NF‐ĸB signaling pathway

**Fig. 9:**
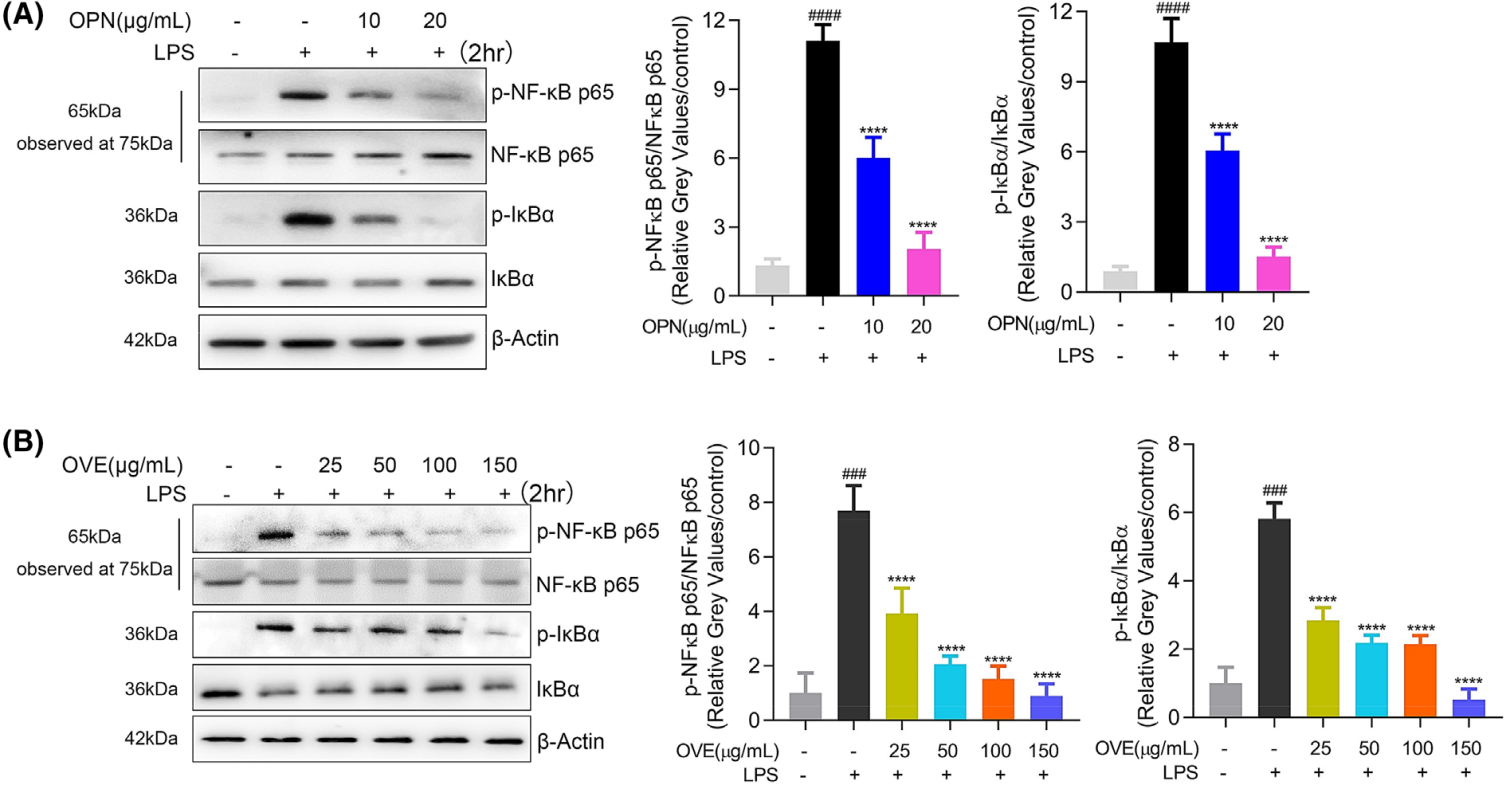
Effects of OPN and OVE on activation of NF‐ĸB pathways in LPS‐stimulated RAW264.7 cells. Cells were pretreated with OVE or OPN at different concentrations for 1 h. Expression levels of p‐NF‐ĸB p65, NF‐ĸB p65, p‐IĸBα, and IĸBα were detected after 24 h of LPS treatment. (A) OPN treatment. (B) OVE treatment. All experiments were carried out in triplicates and data are presented as means ± SDs; one‐way ANOVA analysis was adopted for multiple comparisons; ^###^ *P* < 0.001, ^####^ *P* < 0.0001, compared to the untreated control group; ****P < 0.0001, compared to the LPS control group.

To verify the predicted mechanisms of OPN in inflammation inhibition, we detected NF‐ĸB p65 and IĸBα phosphorylation in RAW264.7 cells after 2 h of LPS stimulation. OVE treatment was employed in this setting for further confirmation. As a result, activation of these two signaling transducers was significantly decreased with OPN (Fig. 9A) and OVE (Fig. 9B), implying the involvement of NF‐ĸB signaling pathways in the anti‐inflammation effects of OPN and *O. vulgatum*.

#### Effects of OPN on MAPK signaling pathways

**Fig. 10:**
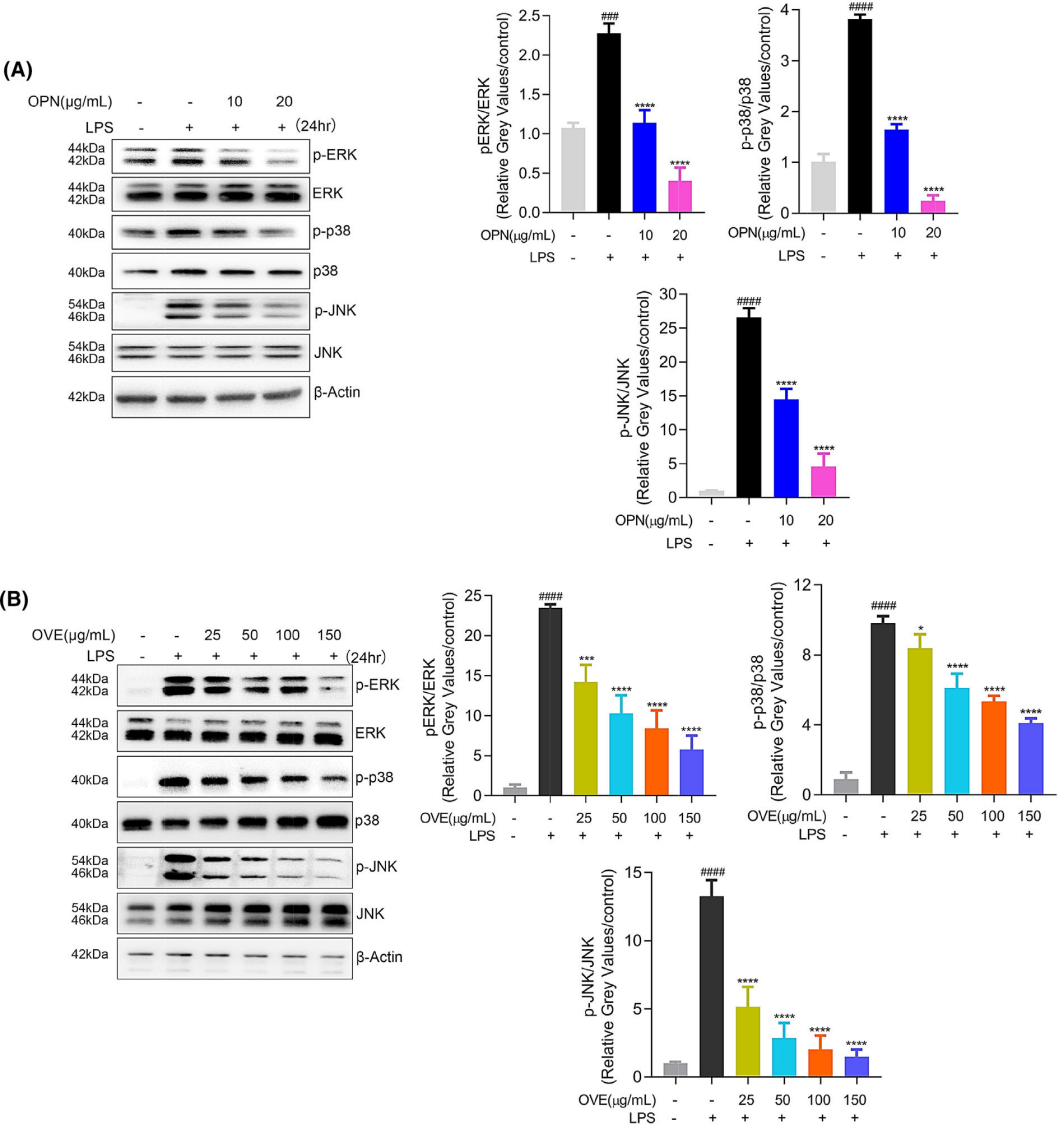
Effects of OPN and OVE on activation of MAPK signaling pathways in LPS‐stimulated RAW264.7 cells. Expression levels of p‐ERK, ERK, p‐p38, p‐38, p‐JNK, and JNK were detected in the same samples for COX‐2 detection after 24 h of LPS stimulation. (A) OVE treatment. (B) OPN treatment. All experiments were carried out in triplicates and data are presented as means ± SDs; one‐way ANOVA analysis was adopted for multiple comparisons; ^###^ *P* < 0.001, ^####^ *P* < 0.0001, compared to the untreated control group; ***P < 0.001 and ****P < 0.0001, compared to the LPS control group.

Additionally, activating specific MAPK signaling transducers, including ERK, p38, and JNK, are also known to induce the expression of inflammatory mediators. To determine the involvement of these transducers, RAW264.7 cells were stimulated with LPS for 24 h and OVE treatment was included in these tests as confirmatory proof. As presented in Fig. 10A,B, OPN and OVE pretreatment significantly decreased ERK, p38, and JNK phosphorylation levels, confirming the involvement of MAPK signaling pathways in the anti‐inflammation effects of OPN.

## Discussion

Significant progress has been made in understanding the mechanisms underlying the development of inflammation‐related diseases. Researchers have been investigating natural products for inflammation alleviation, and many constituents of natural products were identified as potential drugs for clinical studies [34, 35]. The genus *Ophioglossum* is an ancient fern that belongs to the family *Ophioglossaceae*. Over 50 species in this genus have been reported globally, with *O. vulgatum*, *O. pedunculosum*, *O. petiolatum*, *O. thermale*, and *O. reticulatum* being the most widely distributed in China [24, 25]. This study focused on the pharmacological activity of *O. vulgatum* against inflammation. Our data demonstrated the anti‐inflammatory properties of *O. vulgatum* and confirmed that OPN is one active component responsible for the anti‐inflammatory effect using the bioactivity‐guided fractionation method. Our further assessments finally demonstrated the inhibitory effect and the mechanism of action of OPN in inflammation control.

Consistent with a previous study on the anti‐inflammation activity of *O. thermale* extracts, our initial screening clearly presented that EA layer of OVE has the highest inhibitory effect on inflammation, supporting more exploration of compounds in EA fractions [28]. Flavonoids, which consists of a heterocyclic pyran or pyrone ring and two benzene rings, are the active ingredients for many herbal medicines [36, 37]. It is predicted that the medicinal properties of *Ophioglossum* species are significantly associated with their high flavonoid content [38]. Herein, our screening identified DHQ, quercetin, and luteolin as being consistent with the findings of the previous phytochemical analysis. As the most abundant dietary flavonoids, quercetin and luteolin are known for their beneficial health effects, including anti‐inflammatory, antiviral, antimycobacterial, hepatoprotective, and even anticancer properties [39, 40, 41, 42]. DHQ, also known as taxifolin, is used as a food supplement in commercial products and as a chemotherapeutic agent because of its widespread bioactivity [43, 44]. A recent study has reported the protective role of taxifolin against COVID‐19 [43]. 3MQ, a common methylated derivative of quercetin, is an effective antioxidant that alleviates LPS‐induced oxidative stress. Furthermore, it has been reported that 3‐*O*‐methyl flavonoids are the dominant components in all three ferns of *O. vulgatum*, *O. pedunculosum*, and *O. thermale* [45, 46]. Zhang et al. isolated 3MQ from the antioxidative EA fractions of *O. thermale* [28]. Consistent with the findings above, we found that 3MQ is the primary constituent in the highly active fractions of our screening, which indicates the potential application of *O. vulgatum* in anti‐inflammatory treatment.

OPN belongs to the homoflavonoid family, a special type of flavonoids characterized by holding one additional carbon directly attached to the classical C6‐C3‐C6 backbone. Previous studies found that homoflavonoids are abundant in *Ophioglossum* and thus have chemotaxonomic significance for this genus [33, 47]. To date, 14 homoflavonoids and homoflavonoid glucosides have been isolated and classified into two types based on the fragmentation characteristics using electrospray ionization multistage mass spectrometry [33, 47, 48, 49]. Type I homoflavonoids, represented by ophioglonol and its glucoside forms, are characterized by one more carbon atom at the C‐3 position of the C ring. Type II homoflavonoids, including OPN, are characterized by one extra carbon atom at the C‐2′ site of the B‐ring, resulting in the formation of another ring D (Fig. 3). In the exploration of pharmacological functions, Lin et al. reported that OPN has anti‐HBV surface antigen activity, indicating its potential as a therapeutic agent for hepatitis [33]. In the present study, OPN was isolated from fraction EA4‐7, and, to the best of our knowledge, this is the first extensive study on its anti‐inflammatory activity using *in vitro* and *in vivo* models.

Besides, in the comparative analysis of the isolated compounds, our data revealed the high effectiveness of quercetin and 3MQ on NO production, consistent with the findings of previous studies [50, 51]. However, OPN is as effective as DHQ and luteolin in NO inhibition and cell safety. Consequently, further investigations of OPN in viral infections, cancer therapy, and functional foods would be desirable to reveal its additional potentials in human health care.

The peritoneum is a convenient source of cells to obtain macrophages. Injection of eliciting agents, such as sterile thioglycolate, could increase the yield of ‘inflammatory’ macrophages from the peritoneum (PEMs) for biomedical research. It is widely accepted that PEMs display higher heterogeneity than BMDMs and may function differently from resident cells [52, 53]. Therefore, this could explain the observed differences in response to OPN between PEMs and BMDMs.

This study used LPS, a bacterial polysaccharide, as the stimulus for inflammatory responses. As expected, LPS significantly induced NO, IL‐1β, IL‐6, and TNFα synthesis in RAW 264.7 cells and primary cultures of murine macrophages [54]. LPS is known to act on the inflammatory cascade via binding to the TLR4 complex and ultimately promoting transcription of pro‐inflammatory genes via activating NF‐ĸB and MAPK pathways [55]. NF‐κB, an essential transcription factor from the NF‐κB/Rel protein family, is suppressed by the inhibitory proteins, such as IκB family member IκBα [56]. When inflammatory signals bind to the receptors on the cell membrane, IκBα is phosphorylated and degraded [57], releasing the NF‐κB dimer into the nucleus. This promotes the transcription process of pro‐inflammatory mediators with NF‐κB binding sites. In addition, accumulating evidence demonstrates that extracellular stimuli can activate MAPK pathways and three conventional groups of cascades, extracellular signal‐regulated kinase (ERK), Jun‐amino‐terminal kinase (JNK), and p38 MAPK, regulate the production of pro‐inflammatory cytokines at the transcriptional and post‐transcriptional levels [13]. Accordingly, the observation that OVE and OPN dose‐dependently inhibit the gene expression levels of pro‐inflammatory cytokines provided clues for investigating the status of these two canonical signaling pathways. Consequently, our data confirmed that *O. vulgatum* and OPN demonstrated anti‐inflammatory activity in response to LPS by deactivating NF‐ĸB and MAPK pathways. In future, broad tests with other pro‐inflammatory stimuli, such as cytokines (IFNγ, TNFα, and IL‐1β) and virus infection, are suggested. In addition, using *O. vulgatum* and OPN in other inflammation‐related scenarios, such as skin inflammation, hepatitis, neuroinflammation, obesity, and cancer, should be explored.

## Conclusion

This study demonstrated the antioxidant and anti‐inflammatory properties of *O. vulgatum* and confirmed its potential in alleviating inflammatory diseases. Our bioassay‐guided screening revealed the highly active constituents of *O. vulgatum* in inflammation inhibition. Additionally, we validated the anti‐inflammatory effects of OPN using *in vitro* and *in vivo* models, further promoting its practical application as a natural compound for treating inflammation‐related disorders. In future, clinical validations, including clinical trials, are required to confirm the anti‐inflammatory potential of OPN.

## Conflict of interest

The authors declare no conflicts of interest.

### Peer review

The peer review history for this article is available at https://www.webofscience.com/api/gateway/wos/peer‐review/10.1002/2211‐5463.13914.

## Author contributions

XZ, CT, DY, SL, and XH conducted the experiments and obtained the data. JL, YC, and XH confirmed the authenticity and the analysis of the data. XZ, CT, and XH wrote the manuscript. All authors have read and agreed to the published version of the manuscript.

## Data Availability

Data used for compound isolation and analysis are available on reasonable request from the corresponding author.
